# Clinical outcomes of Preserflo MicroShunt implant procedure in open‑angle glaucoma patients - a 1000 eyes case-series; including subanalysis of combined cataract-glaucoma surgery and standalone surgery in pseudophakic eyes

**DOI:** 10.1007/s00417-026-07199-z

**Published:** 2026-03-28

**Authors:** Karsten Klabe, Jan Philipp Werth, Hakan Kaymak, Andreas Fricke

**Affiliations:** 1 Internationale Innovative Ophthalmochirurgie, Martin-Luther-Platz 22/26, Düsseldorf, 40212 Germany; 2 Gottfried O.H. Naumann-Institut für Epidemiologie und Prävention der Myopie, Universitätsklinikum des Saarlandes, Augenklinik / Gebäude 22, Kirrberger Straße 100, Homburg/Saar, 66421 Germany

**Keywords:** Glaucoma, MIBS, MicroShunt, Postoperative Complications, Combined Glaucoma Surgery

## Abstract

**Purpose:**

This retrospective, single-center study aimed to determine the long-term real-world efficacy and safety of the MicroShunt over up to a 6-year period in a heterogeneous glaucoma population of 1001 eyes: including pseudophakic eyes and eyes undergoing combined cataract and MicroShunt surgery.

**Methods:**

Data were analyzed from 1001 eyes implanted with the MicroShunt between January 2019 and May 2025. The outcomes collected were intraocular pressure (IOP) reduction, complete and qualified surgical success at targets of 6–14, 6–18, and 6–21 mmHg, the medication burden, and the postoperative complications. Subgroup analyses compared all the eyes implanted with pseudophakic eyes or those undergoing combined cataract and MicroShunt surgery.

**Results:**

The mean baseline IOP was 24.8 ± 8.4 mmHg across all eyes. IOP decreased to 9.6 ± 4.4 mmHg on postoperative day 1 and remained significantly reduced at 1 year (13.2 ± 3.9 mmHg) and 6 years (13.9 ± 2.3 mmHg). The IOP trajectories were similar in the subgroups. The IOP was slightly lower in eyes that had combined surgery. At 5 years, the complete success was 54%, 36%, and 8% for IOP thresholds of 6–21, 6–18, and 6–14 mmHg, respectively; while the qualified success rates were 62%, 38%, and 15%, respectively. The number of medications decreased significantly after surgery. Early postoperative complications were infrequent and usually transient. The complications were similar across subgroups with choroidal detachment (≤ 12%) and microhyphaema (10–15%) being the most common. Late complications occurred less often, and device-related failure leading to secondary glaucoma surgery rarely occurred (≤ 4%).

**Conclusion:**

The MicroShunt provided a sustained reduction in IOP and medication burden with a low incidence of complications over the follow-up period. The outcomes in pseudophakic eyes and those undergoing combined cataract surgery were like the cumulative population. The inclusion of pseudophakic and combined surgery eyes reflects the real-world situation in glaucoma practice. These results build on mid-term evidence to support the reliability and safety of the MicroShunt.

**Supplementary Information:**

The online version contains supplementary material available at https://doi.org/10.1007/s00417-026-07199-z.

## Abstract (highlights)

**Key messages:**

*****What is known***:**

- The implantation of the MIBS device (Preserflo Microshunt) can be performed as stand-alone procedure or in combination with cataract surgery. For both procedures, success in lowering IOP and reducing medication burden has so far only been demonstrated in clinical studies and real-life publications with small numbers of patients, but not with large real-life data sets.

*****What is new***:**

- This publication presents large-scale real-world data on the use of MicroShunt in over 1000 eyes as a standalone procedure or in combination with cataract surgery in a single glaucoma center.
- Pseudophakic eyes and those undergoing combined cataract and MicroShunt surgery demonstrated comparable IOP and medication reduction over several years.
- The complication rates were similar between the different procedures. Device-related failure requiring secondary glaucoma surgery was rare, and the overall safety profile of this real-life observation does not differ from data from clinical trials.

**Supplementary Information:**

The online version contains supplementary material available at https://doi.org/10.1007/s00417-026-07199-z.

## Introduction

The treatment of glaucoma has progressed, nonetheless it remains one of the leading causes of blindness worldwide, ranking only second to cataract [1, 2]. Intraocular pressure (IOP) reduction is the mainstay of glaucoma treatment [3, 4]. In glaucoma cases, IOP reduction is initially achieved via pharmacological or selective laser treatment, unless the patient’s IOP cannot be reduced sufficiently to prevent the risk of the progression to sight loss, there is a risk of optic nerve damage, a progression of visual field defect, or medication intolerance [3, 5].

In cases of advanced glaucoma, glaucoma surgery with pharmacological augmentation is typically recommended [6]. In terms of surgical IOP control, trabeculectomy has long been considered the gold standard of treatment. Trabeculectomy creates a small excision of the trabecular meshwork and/or corneoscleral tissue with the aims of creating drainage of the aqueous humor from within the eye to below the conjunctiva where it can effectively be absorbed.

Despite the efficacy of trabeculectomy, the procedure is associated with complex post-operative care and a substantial risk of early and late complications; including an increased incidence of ocular hypotony, the occurrence of a shallow anterior chamber, an increased risk of hyphema, bleb-related infections, and endophthalmitis [6]. The problems associated with trabeculectomy led to the development of minimally invasive glaucoma/bleb surgery (MIGS/MIBS), with the aim of providing safer, more predictable surgical outcomes while reducing surgical trauma and improving the post-operative burden of care [2, 6–8].

The Preserflo™ MicroShunt (Santen Pharmaceutical Co. Ltd, Osaka, Japan) is one such MIBS device. It works by draining aqueous humor from the anterior chamber to the sub-Tenon’s space via a flexible poly(styrene-block-isobutylene-block-styrene) (SIBS) MicroShunt. Early comparative evidence indicates that like trabeculectomy the MicroShunt can also effectively lower IOP and is safe [9–12]. Indeed, the limited evidence suggests that due to the lower incidence of post-operative complications or reduced requirement for reintervention the MicroShunt has a better safety profile. Nonetheless, most of the reports currently available have a limited follow-up period, there are few long-term data [13].

Moreover, glaucoma surgery is often necessitated in a diverse patient population; including patients that have already undergone cataract surgery and have pseudophakic eyes or those with cataract that require lens extraction parallel to glaucoma surgery. Thus, in routine clinical practice, the surgical path depends not only on the least invasive surgery and greatest IOP reduction, but also on the lens status, visual function, and the effectiveness of addressing both conditions simultaneously. These subgroups reflect common real-world epidemiological patterns in present-day glaucoma surgery and affect differences in early and late complications and durability of surgical success. Understanding outcomes in the real-world clinical context is therefore essential to fully characterize the long-term performance of subconjunctival drainage devices such as the Preserflo™ MicroShunt.

Thus, the present study sets out to present large scale real- world evidence over six years of MicroShunt use from over 1000 eyes from a single glaucoma office. The study aimed to quantify the long-term IOP control and medication reduction that could be achieved using the MicroShunt; to determine the rates of complete and qualified success under different IOP targets; and describe the occurrence of postoperative complications. In particular, the effectiveness of the MicroShunt in pseudophakic eyes is analyzed, distinguishing between implantation during combined cataract-glaucoma surgery and as a standalone procedure. The data should contribute detailed insight into the sustained efficacy and safety of the MicroShunt and its contribution to glaucoma surgery.

## Methods

### Patients and data collection

This was a retrospective, single center cohort observation that analyzed data from patients that were implanted with the Preserflo™ MicroShunt during routine clinic visits from January 2019 to May 2025. All procedures performed were in accordance with the ethical standards of the institutional research committee and with the 1964 Helsinki declaration and its later amendments or comparable ethical standards. In accordance with good clinical practice, all patients gave their consent to undergo glaucoma and cataract surgery. However, given the retrospective design and the exclusive use of anonymized data, individual consent forms were not obtained for this study.

Study participants were categorized into three groups: (1) all eyes: all eyes that have undergone MicroShunt surgery; (2) pseudophakic eyes: eyes which were pseudophakic prior MicroShunt implantation (subgroup of 1); and, (3) combined cataract with MicroShunt surgery: eyes were previously phakic and underwent combined surgery (subgroup of 1). The remaining subgroup in group 1 consists of phakic eyes that underwent a standalone glaucoma surgery. These eyes are not pseudophakic eyes with a MicroShunt and were therefore not explicitly considered.

For inclusion in the study patients had glaucoma with uncontrolled intraocular pressure (IOP) or increased medication burden or insufficient compliance and had at least 6-months of follow-up. The pre-operative data extracted included the patients’ demographic features, glaucoma type and severity categorized according to EGS guidelines (Hodapp-Anderson-Perish criteria) [14]. Visual field mean deviation (Humphrey: SITA 24 − 2; Carl Zeiss Meditec, Jena, Germany), best-corrected visual acuity (BCVA), IOP (Goldmann Applanation Tonometry) without wash-out, number of glaucoma medications, corneal endothelial cell density (Cellcheck; KONAN medical, Irvine, USA), and cup-to-disc (C/D) ratio were recorded. In addition, previous glaucoma treatments including medications, selective laser trabeculoplasty (SLT), and surgical interventions were also documented. All evaluated pre-operative and follow-up observations were performed in our practice.

The primary outcome measures to be determined were the IOP change from baseline, the complete success, the qualified success, the reduction in medications, and the complication rates. Complete success was defined as: IOP control within target ranges (6–14 mmHg, 6–18 mmHg, or 6–21 mmHg) without additional glaucoma medications. Qualified success was defined as IOP control within the target ranges with or without glaucoma medications. Failure was defined as IOP above the upper threshold or below 6 mmHg or the need for additional glaucoma surgery. Eyes requiring secondary glaucoma surgery were censored at the time of the additional procedure in the survival analyses.

### Surgery

All surgeries were performed exclusively by two surgeons (KK, JPW) using standardized techniques for Preserflo™ MicroShunt implantation in accordance with the manufacturer’s instructions (Santen Pharmaceutical Co. Ltd, Osaka, Japan). Surgery was performed as a standalone procedure in phakic of pseudophakic eyes or combined with cataract extraction and intraocular lens implantation. In most cases, at the surgeon’s discretion, the implantation procedure was performed in the supranasal quadrant. The Tenon’s capsule was bluntly dissected to expose the bare sclera and Mitomycin C (0.4 mg/mL, first 131 eyes with 0.2 mg/mL) [15] was applied using soaked swabs to the sub-Tenon’s space for approximately 3 min, before rinsing with balanced salt solution. A 4 mm scleral tunnel was prepared from the blue/gray zone and the MicroShunt inserted through the scleral tunnel into the anterior chamber. The fins were positioned flush with the scleral surface, and the flow of aqueous humor was confirmed through the distal tip. The patency of filtration was verified, and Tenon’s (1.5–2 mm behind limbus) and conjunctiva were adapted separately with absorbable sutures (ONASORB^®^PGA - REF: ON456495, Surgical Specialties México) [16].

### Analyses

The statistical analyses were performed using Microsoft Excel (Microsoft 365, Redmond, WA, USA) and OriginPro 2017 SR1 (OriginLab Corporation, Northampton, MA, USA). Continuous variables are presented as mean ± standard deviation (SD) or median and interquartile range, as appropriate. Categorical variables are summarized as absolute frequencies and percentages.

To determine the significance of changes in IOP, paired sample t-tests were performed to compare the data before and after surgery at various time points. Unpaired t-tests were used to determine differences in IOP between the pseudophakic and the combined surgery subgroup. A Wilcoxon rank sum test and a Mann-Whitney U test were used for between group comparisons on the number of glaucoma medications. An ANOVA analysis was performed to compare subgroups 2, 3, and the remaining subgroup from group 1, which consists of phakic eyes that underwent standalone glaucoma surgery (supplementary material). Eyes requiring secondary surgery other than bleb revision or needling were excluded from further analysis. All statistical tests were two-sided. A p-value of ≤ 0.05 was considered statistically significant.

## Results

### Demographic and clinical features

In the study period a total of 1001 eyes from 673 patients underwent MicroShunt surgery. There were 513 pseudophakic eyes from 378 patients that underwent MicroShunt surgery alone; 97 phakic eyes from 67 patients that underwent combined cataract and MicroShunt surgery (Table 1). The remaining 391 phakic eyes from 255 patients underwent MicroShunt surgery alone.

The mean age of the patients overall was 70 ± 11 years (range 27–95). The mean ages of the sub-groups were similar. Of the 1001 eyes 442 were male (44%) and 559 were female (56%). The participants were mostly Caucasian (97%). The right and left eye were operated on in a similar number of cases. Most patients had primary open-angle glaucoma (69%), or pseudoexfoliative glaucoma (27%). A summary of the patient’s demographic data and clinical characteristics are shown in Table 1.

**Table 1:** Demographic and clinical characteristics of patients/eyes undergoing MicroShunt surgery

	all eyes	pseudophakic eyes	Eyes with combined microshunt and cataract surgery
No. eyes	1001	513	97
No. patients	673	378	67
Gender, male (%)	442 (44%)	200 (39%)	43 (46%)
Age, mean ± SD (range) [years]	70 ±11(27–95)	74 ± 10 (27–95)	74 ± 8 (48–87)
Laterality, OD (%)	492 (49%)	253 (49%)	52 (54%)
Lens status, phakic (%)	488 (49%)	-	97 (100%)
Ethnicity, no. (%)			
Caucasian (central european region)	969 (97%)	497 (97%)	98 (99%)
non-Caucasian	34 (3%)	16 (3%)	1 (1%)
glaucoma classification, no. eyes (%)			
POAG	684 (69%)	328 (64%)	62 (64%)
Pseudoexfoliative glaucoma	270 (27%)	152 (30%)	34 (35%)
Pigmentary glaucoma	24 (2%)	14 (3%)	1 (1%)
Neovascular glaucoma	11 (1%)	8 (1%)	-
Uveitic glaucoma	7 (1%)	6 (1%)	-
Iatrogenic glaucoma	5 (< 1%)	5 (< 1%)	-
glaucoma severity^1^, no. eyes (%)*			
eyes with previously visual field test:	864	433	89
early	367 (42%)	140 (32%)	40 (44%)
moderate	182 (21%)	99 (23%)	20 (22%)
advanced	315 (37%)	193 (45%)	29 (33%)
glaucoma visual field, MD ± SD (range)	-10.4 ± 9.0	-12.3 ± 9.0	-10.6 ± 9.3
[dB]	(+ 2.3 to -32.4)	(+ 2.0 to -32.4)	(+ 2.3 to -31.7)
BCVA, MD ± SD			
[logMAR]	0.21 ± 0.27	0.25 ± 0.29	0.29 ± 0.24
corneal endothelial cell density, MD ± SD (range) [cells/mm²]	2382 ± 371 (400–2968)	2296 ± 421 (400–2968)	2438 ± 313 (1477–2950)
C/D ratio, MD ± SD	0.75 ± 0.22	0.77 ± 0.22	0.73 ± 0.23
MMC concentration during surgery			
(soaked swabs), no. eyes (%)			
0.2 mg/mL	131 (13%)	58 (11%)	12 (12%)
0.4 mg/mL	870 (87%)	455 (89%)	85 (88%)

^1^according to visual field defects (early: MD ≤ 6 dB; moderate: 6 > MD ≤ 12 dB; advanced: > 12 dB); POAG-primary open-angle glaucoma; BCVA-best distance corrected visual acuity; C/D: cup-to-disc; MD-mean deviation; SD-standard deviation

*visual field test was not always performed prior Preserflo MicroShunt implantation (surgery performed due to referral from a referring physician or patients lack of visual acuity)

The previous surgical interventions varied according to the subgroup. As shown in Table 2, prior to MicroShunt surgery, most eyes had some form of previous glaucoma medication. Pseudophakic eyes tended to be treated with medication more often than the eyes about to undergo cataract and MicroShunt surgery combined. Most eyes did not have laser trabeculoplasty or any previous surgical treatment. In the “all eyes” group, the majority had no prior glaucoma surgery (71%); whereas a smaller number of eyes had one previous procedure (20%) or more than one procedure (9%). Overall, eyes with previous conjunctival involvement, such as trabeculectomy, canaloplasty, or Xen implantation accounted for 18% of the total 1001 cases; but a greater proportion of pseudophakic eyes than eyes undergoing cataract and MicroShunt surgery combined. MIGS procedures were performed prior to MicroShunt surgery in 8% of all eyes. Again, the pseudophakic subgroup tended to undergo MIGS procedures more often than the combined treatment subgroup.

**Table 2:** Previous glaucoma treatment

	all eyes	pseudophakic eyes	combined microshunt with cataract surgery
Medication classes*, no. eyes (%)			
no medication	67 (7%)	35 (7%)	14 (14%)
prostaglandin analogs	742 (74%)	364 (71%)	62 (68%)
β-receptor antagonists	602 (60%)	320 (62%)	42 (47%)
carbonic anhydrase inhibitors	672 (67%)	340 (66%)	62 (63%)
alpha-2 adrenergic agonists	362 (36%)	175 (34%)	33 (34%)
Rho kinase inhibitors	27 (3%)	17 (3%)	4 (4%)
parasympathomimetics	14 (1%)	4 (1%)	-
SLT, no. eyes (%)	291 (29%)	156 (30%)	17 (18%)
Prior surgical glaucoma treatment, no. eyes (%)			
none	708 (71%)	289 (56%)	95 (96%)
1	205 (20%)	155 (30%)	2 (2%)
> 1	90 (9%)	71 (14%)	2 (2%)
Eyes with at least one glaucoma surgery with previous conjunctival involvement, no. eyes (%)			
(Canaloplasty ab externo / Trabeculectomy / Xen implant)	185 (18%)	137 (27%)	3 (3%)
Eyes with at least one angle-based MIGS-procedure, no. eyes (%)	80 (8%)	70 (14%)	1 (1%)

*SLT* Selective Laser Trabeculoplasty, *MIGS* Minimally Invasive Glaucoma Surgery

*Combination agents were frequently used in treatment

After two years the endothelial cell density changed slightly by 1% from 2482 ± 290 to 2452 ± 292 (data available for only 144 eyes at baseline and after 2 years) for all eyes, from 2425 ± 325 to 2396 ± 316 (66 eyes) for previously pseudophakic eyes and from 2532 ± 156 to 2533 ± 107 (16 eyes) for eyes that had undergone combined surgery.

### Intraocular pressure

**Fig. 1:**
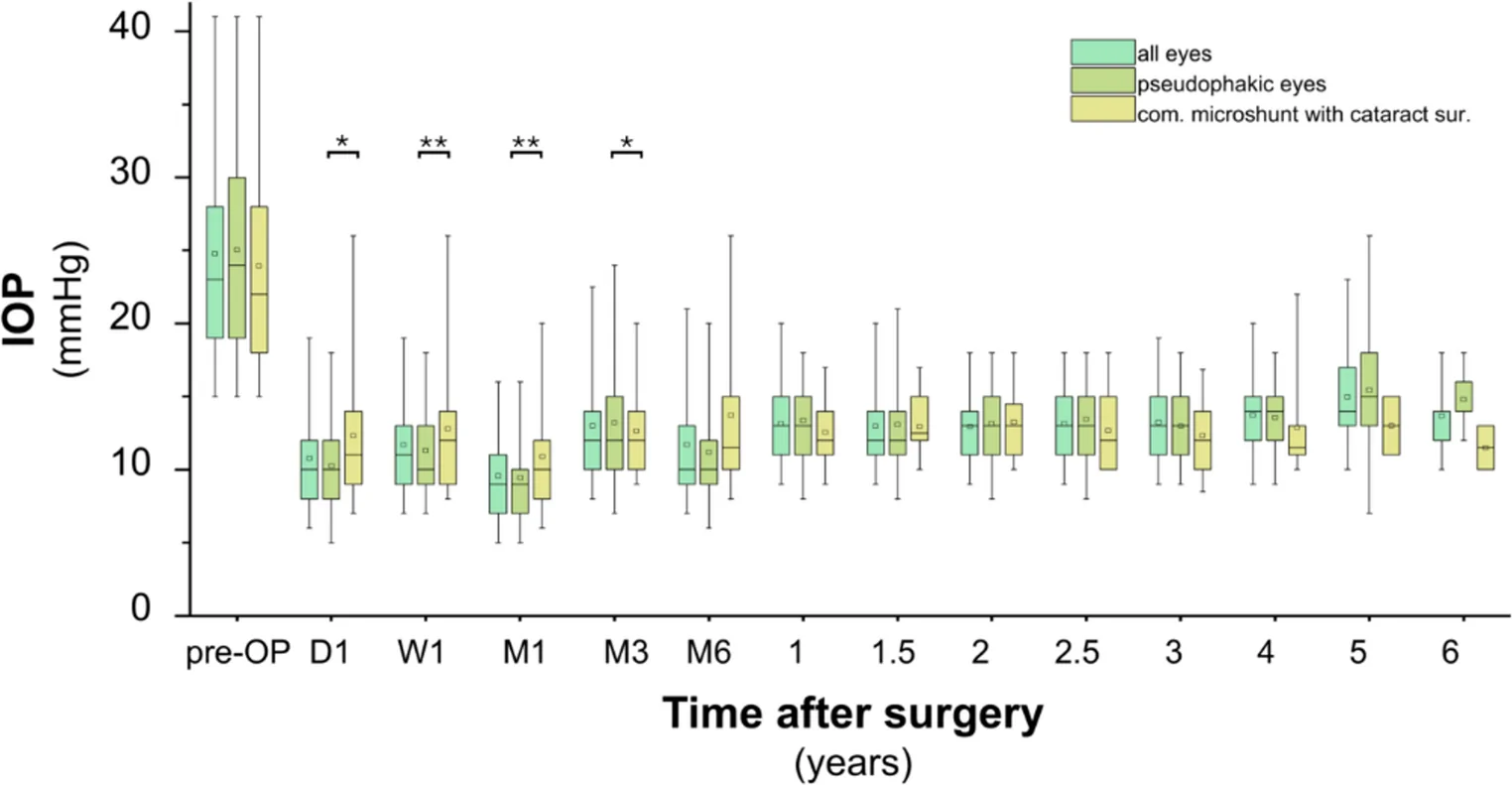
Mean and median medicated intraocular pressure (horizontal black line and square) and percentiles (boxes-quartiles = 25/75; whiskers = 5/95) given preoperatively and across the follow-up period of 6 years. Asterisk denotes statistical significance between pseudophakic and combined surgery -determined by Student t-Test (* <0.05; ** <0.001)

Figure 1 shows the mean IOP and number of medications over the 6-year follow-up. The mean IOP pre-operatively was 24.8 ± 8.4 mmHg across all eyes, 25.0 ± 8.5 mmHg in pseudophakic eyes and 24.0 ± 8.1 mmHg in eyes that had combined surgery (Supplementary Table 1). Post-operative day 1, the mean IOP had already fallen to 9.6 ± 4.4 mmHg in all eyes (median 9 mmHg), 9.6 ± 4.7 mmHg (median 9 mmHg) in pseudophakic eyes, and 10.8 ± 5.0 mmHg (median 10 mmHg) in eyes that had undergone combined surgery. Compared to the pre-operative IOP this was a significant improvement in all cases.

In the long-term, MicroShunt also controlled IOP over the follow-up period. At 1 year, the mean IOP was 13.2 ± 3.9 mmHg (median 13 mmHg) in all eyes, 13.4 ± 3.6 mmHg (median 13 mmHg) in pseudophakic eyes, and 12.6 ± 2.5 mmHg (median 12 mmHg) in eyes undergoing combined surgery.

Similarly, in 4 years, there was a sustained reduction in IOP. The mean IOP was 13.7 ± 3.1 mmHg (median 14 mmHg) in all eyes, 13.6 ± 3.1 mmHg (median 14 mmHg) in pseudophakic eyes, and 12.9 ± 3.4 mmHg (median 12 mmHg) in eyes undergoing combined surgery (Fig. 1).

**Fig. 2:**
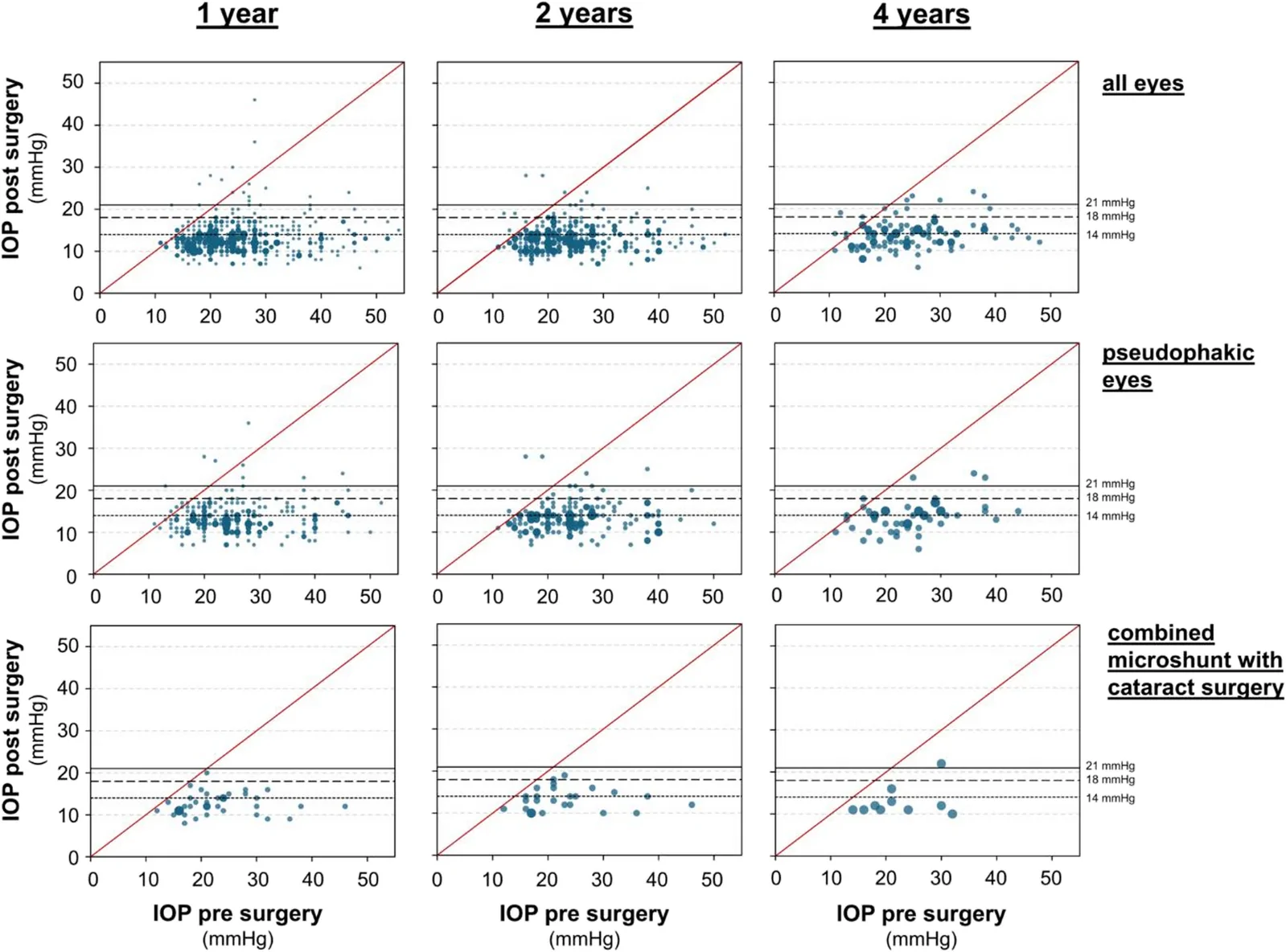
Scatter plot of preoperative IOP versus postoperative IOP at 1 year (433 eyes), 2 years (323) and 4 years (109): The size of the bubbles indicates the number of eyes for that data point. Points below the diagonal line represent a decrease in IOP compared to the preoperative data

The analysis of the pre-operative IOP compared to the post-operative IOP confirmed that this pressure reduction was consistent relative to baseline IOP levels in all groups (Fig. 1b). Most eyes achieved a post-operative IOP that was below the target thresholds of 14, 18, and 21 mmHg, i.e., most data points were below the line of equivalence in the scatter plot (45° line Fig. 2). The pre- versus post-operative IOP analysis also indicated that the reductions were similar between surgical groups. Comparing groups, the data showed that pseudophakia subgroup had significantly lower IOP values than the combined group in the first six months after surgery; thereafter, there was no longer any significant difference between the subgroups (Suppl. Table 1). The same applies to the comparison with phakic eyes, in which the implantation of the MicroShunt was performed as a standalone procedure (Suppl. Table 2).

The scatter plots also showed that eyes with higher pre-operative IOPs showed a tendency towards a greater absolute reduction because their post-operative pressures fell much further below the line of equivalence, whereas those with lower baseline IOPs maintained stable control within target ranges because their IOP, which was already closer to the target thresholds, had less capacity for a further reduction.

The complete data set on the IOP is shown in supplementary Table 1.

### Surgical Success Rates

**Fig. 3:**
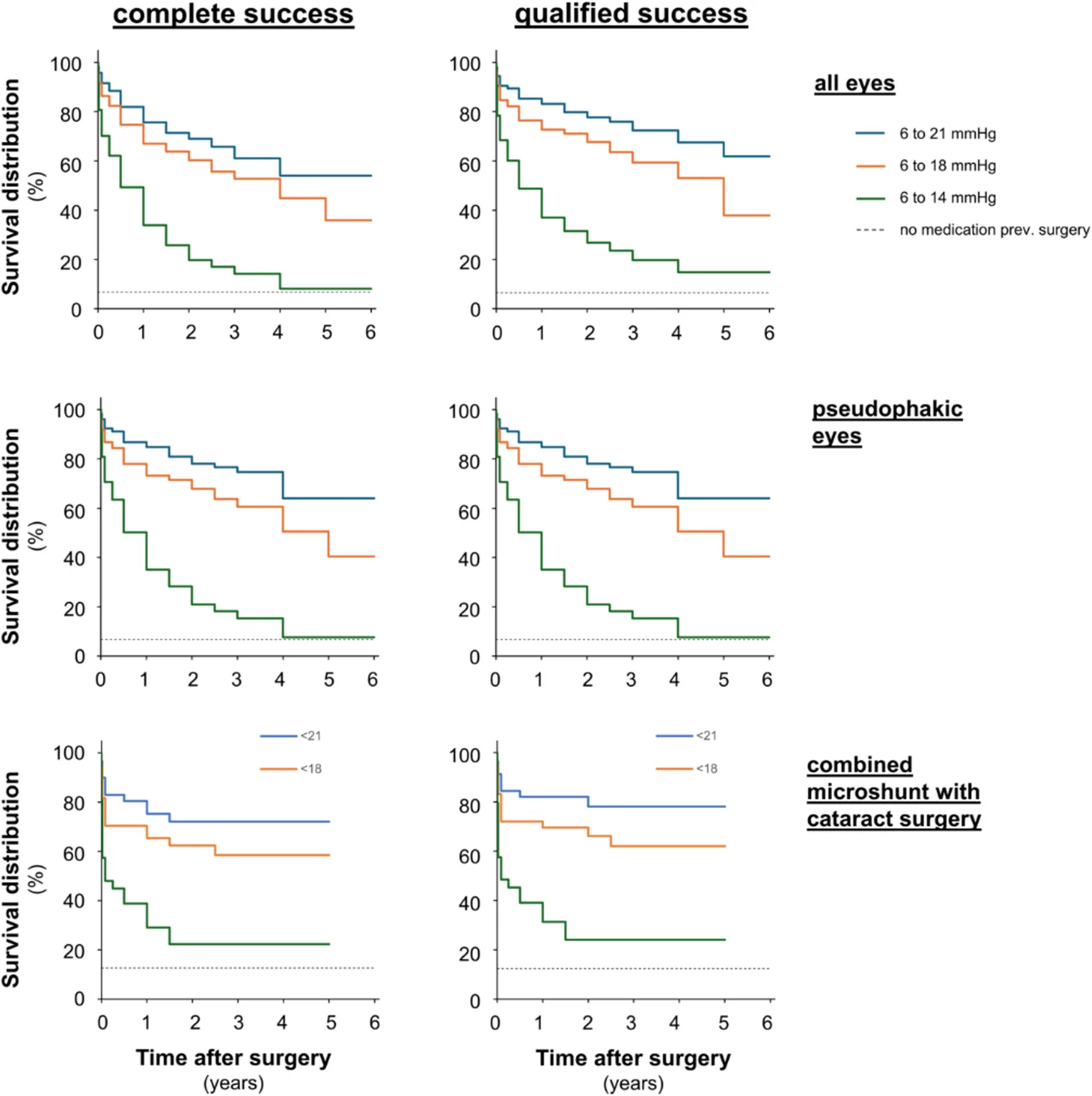
Kaplan-Meier survival curves for complete and qualified success within the IOP ranges of 6 to 14 mmHg, 6 to 18 mmHg and 6 to 21 mmHg

Kaplan–Meier survival curves for complete and qualified success are shown in Fig. 3. The curves show that success rates varied based on the target IOP criteria.

The cumulative rate of complete success (without medications) for group 1 at 4 years was 54% when using an IOP target of 6–21 mmHg, 45% with a target of 6–18 mmHg, and 8% with a target of 6–14 mmHg.

When qualified success (with or without medications) was considered, the corresponding rate at 4 years was 68% for the 6–21 mmHg target, 53% for the 6–18 mmHg target, and 15% for the 6–14 mmHg target. The group with combined surgery had better success rates compared to the pseudophakic subgroup (Fig. 3).

Cumulatively, the data showed that in both complete and qualified success the more restrictive the IOP target, the poorer the survival distribution; nonetheless, all types of MicroShunt surgery provide IOP control over the follow-up period.

### Medication burden

There was a significant reduction in the mean number of medications before and after surgery in all eyes (Suppl. Table 1). The pre-operative medication burden was 2.41 ± 1.14 in all eyes, 2.36 ± 1.16 in pseudophakic eyes, and 2.16 ± 1.28 in eyes undergoing combined surgery. Postoperatively, there was overall a marked reduction in the medication requirements. At 1 week, the mean number of glaucoma medications was 0.01 ± 0.12 in all eyes, 0.01 ± 0.09 in pseudophakic eyes, and 0.04 ± 0.25 in eyes undergoing combined surgery. At 1 year, the mean number of medications was 0.13 ± 0.54 in all eyes, representing a statistically significant reduction relative to the pre-operative number.

**Fig. 4:**
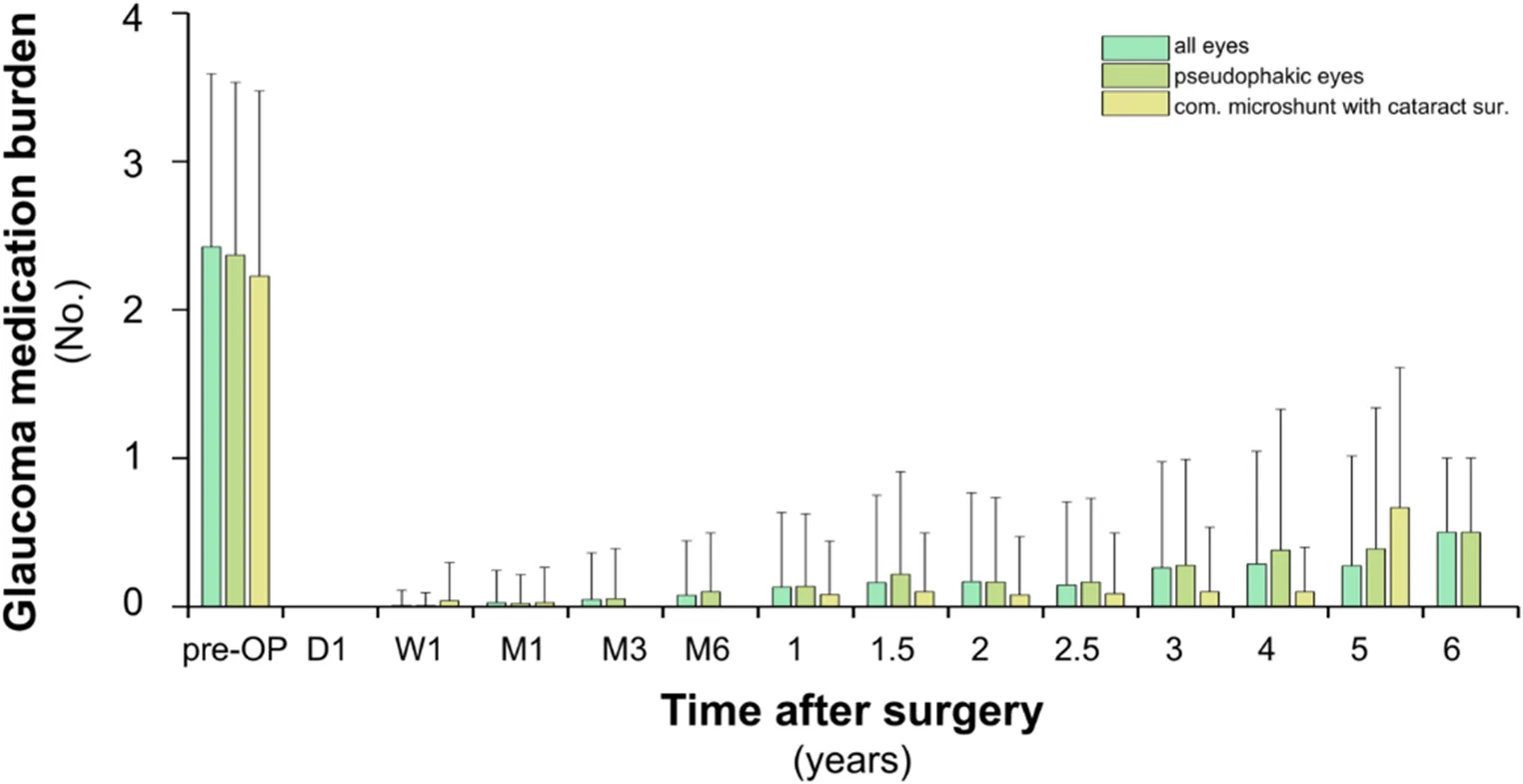
Mean number of glaucoma medications with standard deviation (+ SD) given preoperatively and across the follow-up period of 6 years. There was no statistical significance between pseudophakic and combined surgery -determined by Wilcoxon signed rank test

By 4 years the values increased marginally in each sub-group with reference to the first week. The mean number of medications was then 0.29 ± 0.76 in all eyes, 0.39 ± 0.96 in the pseudophakic eyes, and 0.10 ± 0.30 (median 0) in eyes undergoing combined surgery. Nonetheless, the mean number of medications remained significantly less relative to baseline in all groups. By 6 years there were two few eyes for reliable analysis of the effect. In general, a sustained reduction in the burden of medications in all groups was observed across the follow-up period (Fig. 4).

The complete data set on the number of glaucoma medications is shown in supplementary Tables 1 and 2.

### Complication rates

Most complications occurred in the early postoperative period (≤ 3 months) (Table 3). In all eyes, the most common early complications were microhyphaema in 15%, choroidal detachment in 12%, visible hyphema in 11%, and corneal changes such as Descemet’s folds in 4%, and epithelial edema in 3%. Similarly, the most common early complications in pseudophakic eyes were microhyphaema in 13%, choroidal detachment in 12%, visible hyphema in 7%, Descemet’s folds in 3%, and corneal epithelial edema in 4%. In the combined surgery group, there were microhyphaema in 10%, choroidal detachment in 10%, visible hyphema in 7%, Descemet’s folds in 7%, and corneal epithelial edema in 3%.

Late complications (> 3 months) were less frequent (Table 3). In all eyes, the most common late events were cystoid macular edema in ≤ 7%, corneal epithelial edema in ≤ 5%, and Descemet’s folds in 3%. In pseudophakic eyes, the most common late complications were cystoid macular edema, whereas in the combined surgery subgroup, cystoid macular edema and Descemet’s folds were both reported most frequently.

In both the early and late postoperative cases, the incidence of device-related events was low. Overall, malposition was reported in only 2% of eyes, and migration and obstruction in 1% of all eyes. Less than 4% of patients with the MicroShunt required a secondary surgery over the follow-up period regardless of the glaucoma type (Supplementary Table 3); and the frequency of secondary surgery was similar between the surgical subgroups.

During postoperative management, bleb revision was performed in 8.6% of eyes and needling in 16.8%. However, there is a statistically significant difference between the group with pseudophakic standalone and the group with combined surgery in terms of needling-treatment (14.6% vs. 24.7%; *p* = 0.01), but not in terms of bleb revisions (8.2% vs. 6.2%; *p* = 0.50).

**Table 3:** Postoperative complications

complications	all eyes	pseudophakic eyes	combined microshunt with cataract surgery
**Early postoperative**, no. eyes (%) (up to 3 months postoperative) *Eyes seen on-site*	631 (63%)	440 (88%)	82 (85%)
Microhyphaema	97 (15%)	65 (15%)	8 (10%)
Visible-Hyphaema	67 (11%)	30 (7%)	6 (7%)
Clinical Hypotony (< 6 mmHg ass. with chor. detachment)	18 (3%)	13 (3%)	1 (1%)
Mild AC depth reduction	18 (3%)	8 (2%)	1 (1%)
Anterior synechiae	9 (1%)	8 (2%)	-
Choroidal detachment	73 (12%)	53 (12%)	8 (10%)
Corneal epithelial edema	17 (3%)	19 (3%)	2 (2%)
Corneal abrasion	23 (4%)	15 (3%)	1 (1%)
Descemet’s folds	27 (4%)	16 (4%)	6 (7%)
Device: Malposition	12 (2%)	5 (1%)	1 (1%)
Device: Migration	2 (< 1%)	1 (< 1%)	1 (1%)
Device: Obstruction	10 (2%)	4 (1%)	2 (2%)
Cystoid macular edema	20 (3%)	14 (3%)	2 (2%)
Endophthalmitis	1 (< 1%)	1 (< 1%)	-
**Late postoperative**, no. eyes (%) (after 3 months postoperative) *Eyes seen on-site*	597 (60%)	289 (58%)	56 (58%)
Microhyphaema	6 (1%)	3 (1%)	-
Clinical Hypotony (< 6 mmHg ass. with chor. detachment)	2 (< 1%)	2 (1%)	-
Mild AC depth reduction	7 (1%)	2 (1%)	-
Anterior synechiae	5 (1%)	4 (1%)	-
Choroidal detachment	10 (12%)	6 (2%)	3 (5%)
Corneal epithelial edema	19 (3%)	13 (4%)	-
Corneal abrasion	3 (1%)	2 (1%)	-
Descemet’s folds	14 (2%)	9 (3%)	-
Device: Migration	4 (1%)	3 (1%)	-
Device: Obstruction	6 (1%)	2 (1%)	-
Cystoid macular edema	34 (6%)	21 (7%)	3 (5%)
**Postoperative Management ** (1 year postoperative)			
Bleb-Revision, no. eyes (%)	86 (8.6%)	42 (8.2%)	6 (6.2%)
Needling, no. eyes (%)	168 (16.8%)	75 (14.6%)	24 (24.7%)
Needling + Bleb-Revision, no. eyes (%)	25 (2.5%)	9 (1.8%)	3 (3.1%)

*AC* Anterior chamber

## Discussion

This large, heterogeneous glaucoma population study of 1001 eyes showed a decrease in mean IOP over the 6-year follow-up period, indicating that the MicroShunt offers stable IOP reduction in the long term. Pseudophakic eyes and those undergoing combined cataract and MicroShunt surgery demonstrated comparable long-term pressure reduction. The success rates reflected sustained IOP control, with most eyes achieving qualified success over time. The complication rates were similar between treatment groups, and a low medication burden was maintained post-operatively indicating that the device is effective and dependable. Device-related failure requiring secondary glaucoma surgery was rare, and the overall safety profile was favorable, with early complications being mostly transient and late complications infrequent.

These results strengthen evidence on the MicroShunt’s performance from previously published mid-term outcomes showing a reduction in IOP [9, 12, 13, 17, 18]. At postoperative day 1, the mean was approximately 10 mmHg in all eyes, representing an early reduction of around 60% from the baseline value. After 1-year the reduction in IOP observed was greater or commensurate with what is typically reported in the literature. For instance, after 1-year the mean IOP in the present study represented an approximate 47% reduction (24.8 mmHg at baseline to 13.2 mmHg at 1-year), which was like the values reported [17]. Atlas et al. reported 22.1 mmHg at baseline and values down to 14.6 mmHg after surgery, equating to a 35% reduction. This was also like the magnitude of response reported by Jamke et al. [9], which had a reduction from 16.2 mmHg in the median diurnal IOP to 10.5 mmHg, again an approximate 35% reduction using the MicroShunt. The data showed a greater reduction in IOP from baseline than Pillunat et al. [18], who reported a reduction from 14.3 to 16.5 mmHg to 9.5–12.8 mmHg, commensurate with a 22–34% reduction from baseline. This slightly greater reduction herein was an artifact of the higher preoperative IOP in our overall population group. The pseudophakic eyes and those undergoing cataract and MicroShunt surgery combined exhibited comparable long-termreductions.

The overall mean IOP of 13.9 mmHg after 6-years in our study remains on par with the range 3-years post-surgery reported by Atlas et al. (12–13.5 mmHg), indicating that similarly, the MicroShunt is able to deliver sustained IOP control, but beyond the period previously reported. This shows that the performance of the device maintains a reliable reduction in IOP in the long-term.

The magnitude of the IOP reduction was also like those reported using other MIGS devices or other filtering surgeries such as trabeculectomy [9, 17]. In the XEN device, a reduction from 22.1 to 14.4 mmHg (35%) is reported; and in Trabeculectomy 37% (17.6 to 11.1mmHg) after 1-year.

Cumulatively, our study shows a favorable decrease in IOP in the long-term that is similar compared to the other studies. However, the predominance of pseudophakic eyes in our study must be considered when interpreting the outcomes. In other surgical systems, we and other authors have previously reported similar IOP reductions between phakic and pseudophakic eyes [19–21]. Cataract surgery is generally considered beneficial in POAG and primary angle-closure glaucoma because it can improve aqueous humor flow [19, 22], and the cataract surgery on its own may lead to a temporary modest reduction in IOP or reduce lens related variables that one may see in phakic patients such as lens thickening. Nonetheless, the long-term IOP reduction observed in this study is most likely a reflection of the sustained performance of the MicroShunt, however the data aligns well with the real-world epidemiological situation.

Overall, the qualified and complete success further supported the findings of the effectiveness of MicroShunt over time. The qualified success remained high over the follow-up period. Approximately 60–70% of all eyes met the IOP threshold after 6-years. While the threshold for complete success, without medications, was met in approximately 35% of eyes. Compared to eyes that underwent combined cataract and MicroShunt surgery, pseudophakic eyes undergoing standalone surgery showed only a slightly better reduction in IOP in the first three months after surgery. No significant difference was observed in later stages, once again underscoring the suitability of the implant for eyes under both conditions. Similar results have also been reported [23].

The success rates also underscored the importance of the target IOP selection. As expected, stricter thresholds reduced the probability of success, emphasizing the need to individualize surgical goals based on disease severity and risk tolerance. The MicroShunt can therefore serve as a “replacement” for trabeculectomy in advanced cases, and it is more suitable for moderate cases where 15–18 mmHg is acceptable.

Lowering the IOP of course reduced the need for medications; the effect of which was observed across the 6-year follow-up period. The mean number of medications required was 0.5 or less in all the sub-groups. A reduction in medication burden is a clinically relevant benefit in the management of glaucoma and a significant benefit to the patient in the contribution to their quality of life. Likewise, numerous studies support the efficacy of the MicroShunt in reducing the medication burden [3, 12, 24–26].

Not only did the intervention lower the medication burden, but we also observed that in instances where complications occurred, such as micro- or macro-hyphema or choroidal detachment, that these were early transient events that typically resolved without sequelae; and, that the occurrence of late complications were infrequent with the MicroShunt. Poor positioning or obstruction of the MicroShunt were extremely rare events (≤ 2%), and the number of patients requiring a secondary glaucoma surgery due to the inadequate function of the device was 4% or less, which underscores the ease of the surgical procedure and reproducibility in the surgeon’s hands, not to mention the overall safety of the device.

The strengths of this observation are the large sample size, inclusion of a broad glaucoma population reflecting contemporary clinical practice, and the extended observation window of up to 6 years. All surgeries were performed using a standardized technique at a single center, which reduces procedural variability and enhances internal consistency. Several limitations must be acknowledged. First, the retrospective design is subject to selection and information bias, and follow-up was incomplete at later time points, leading to a decreasing number of eyes contributing data beyond 3–4 years. Second, analyses were performed at an eye-level, so potential intra-patient correlation between fellow eyes was not explicitly modelled. Third, for this publication we did not systematically analyze visual field progression, or endothelial cell density, which limits conclusions regarding long-term structural and functional outcomes. This was counterbalanced significantly by the consistent follow-up across thousands of visits which provided robust evidence of the long-term efficacy of the MicroShunt under pseudophakic conditions and in circumstances where cataract surgery was combined with the filtering surgery. The study contributes insight into the sustained efficacy and safety of the MicroShunt and its future contribution to glaucoma surgery.

In conclusion, the real-world analysis of over 1000 patients confirms that the MicroShunt is effective, and offers durable, and safe long-term IOP control and a clinically relevant reduction in medication use. Given that the study included a large number of pseudophakic eyes and a subgroup of eyes undergoing cataract and MicroShunt surgery combined, the evidence contributes to real world data that broadens existing evidence on the MicroShunt beyond the studies available on phakic or single-procedure cases.

## Supplementary Information

Below is the link to the electronic supplementary material.

Supplementary Material 1.

Supplementary Material 2.

Supplementary Material 3.

## Data Availability

The data that support the findings of this study are available from the corresponding author, upon reasonable request. The data are not publicly available due to privacy or ethical restrictions.
