# Supplementary material for: Clinical outcomes of Preserflo MicroShunt implant procedure in open‑angle glaucoma patients - a 1000 eyes case-series; including subanalysis of combined cataract-glaucoma surgery and standalone surgery in pseudophakic eyes
Source: Graefes Arch Clin Exp Ophthalmol. 2026 Mar 28;264(10):3069–80. doi: 10.1007/s00417-026-07199-z (PMC13635017; doi:10.1007/s00417-026-07199-z)
Supplement: Supplementary file 1 [file 417_2026_7199_MOESM1_ESM.pdf]

Supplementary table 1

Intra ocular pressure (IOP) and glaucoma medication: follow-up to 6 years after surgery.

	**all eyes**						**pseudophakic eyes**						**combined microshunt with cataract surgery**							
**Time after surgery**	No. eyes	Median	IOP - mean (mmHg ± SD)	p-Value+	Med. - mean (No.)	p-Value*	No. eyes	Median	IOP - mean (mmHg ± SD)	p-Value+	Med. - mean (No.)	p-Value*	No. eyes	Median	IOP - mean (mmHg ± SD)	p-Value+	Med. - mean (No.)	p-Value*	IOP p-Value++	Med. p-Value**
pre-surgery	1001	23	24.8 (± 8.4)	-	2.41 (± 1.14)	-	513	24	25.0 (± 8.5)	-	2.36 (± 1.16)	-	97	22	24.0 (± 8.1)	-	2.16 (± 1.28)	-	0.19	0.31
1 day	604	9	9.6 (± 4.4)	<0.01	-	-	316	9	9.6 (± 4.7)	<0.01	-	-	60	10	10.8 (± 5.0)	<0.01	-	-	0.04	-
1 week	798	10	10.8 (± 5.0)	<0.01	0.01 (± 0.12)	<0.01	407	10	10.3 (± 4.6)	<0.01	0.01 (± 0.09)	<0.01	77	11	12.3 (± 5.0)	<0.01	0.04 (± 0.25)	<0.01	<0.01	0.14
1 month	788	10	11.6 (± 5.3)	<0.01	0.03 (± 0.25)	<0.01	399	10	11.1 (± 4.7)	<0.01	0.02 (± 0.19)	<0.01	78	11	13.6 (± 8.0)	<0.01	0.03 (± 0.23)	<0.01	<0.01	0.98
1/4 year	707	11	11.7 (± 4.6)	<0.01	0.05 (± 0.36)	<0.01	356	10	11.2 (± 4.1)	<0.01	0.05 (± 0.32)	<0.01	66	12	12.6 (± 4.7)	<0.01	0 (± 0)	<0.01	0.02	0.16
1/2 year	515	12	13.0 (± 4.9)	<0.01	0.07 (± 0.40)	<0.01	253	12	13.2 (± 5.3)	<0.01	0.09 (± 0.38)	<0.01	49	12	12.5 (± 3.4)	<0.01	0 (± 0)	<0.01	0.40	0.07
1 year	422	13	13.2 (± 3.9)	<0.01	0.13 (± 0.54)	<0.01	205	13	13.3 (± 3.6)	<0.01	0.14 (± 0.48)	<0.01	37	12	12.5 (± 2.5)	<0.01	0.08 (± 0.36)	<0.01	0.18	0.43
1.5 years	345	12	13.0 (± 3.4)	<0.01	0.16 (± 0.59)	<0.01	177	12	13.1 (± 3.5)	<0.01	0.22 (± 0.69)	<0.01	29	13	13.0 (± 2.1)	<0.01	0.10 (± 0.40)	<0.01	0.79	0.43
2 years	306	13	12.9 (± 3.0)	<0.01	0.17 (± 0.59)	<0.01	155	13	13.2 (± 3.1)	<0.01	0.17 (± 0.57)	<0.01	24	13	13.3 (± 2.6)	<0.01	0.08 (± 0.39)	<0.01	0.93	0.35
2.5 years	270	13	13.2 (± 2.8)	<0.01	0.14 (± 0.59)	<0.01	134	13	13.4 (± 3.4)	<0.01	0.17 (± 0.56)	<0.01	23	12	12.7 (± 2.7)	<0.01	0.09 (± 0.41)	<0.01	0.28	0.42
3 years	201	13	13.2 (± 3.6)	<0.01	0.25 (± 0.69)	<0.01	92	13	13.0 (± 2.8)	<0.01	0.27 (± 0.71)	<0.01	20	12	12.3 (± 2.5)	<0.01	0.10 (± 0.44)	<0.01	0.33	0.23
4 years	96	14	13.7 (± 3.1)	<0.01	0.29 (± 0.76)	<0.01	40	14	13.6 (± 3.1)	<0.01	0.39 (± 0.96)	<0.01	10	12	12.9 (± 3.4)	<0.01	0.10 (± 0.30)	0.01	0.66	0.53
5 years	40	14	15.2 (± 4.6)	<0.01	0.28 (± 0.74)	<0.01	18	15	16.0 (± 5.8)	<0.01	0.39 (± 0.95)	<0.01	3	13	13.0 (± 1.6)	n/a	0.67 (± 0.94)	n/a	n/a	n/a
6 years	9	14	13.9 (± 2.3)	<0.01	0.50 (± 0.50)	0.02	5	14	14.8 (± 2.0)	n/a	0.50 (± 0.50)	*n/a*	2	12	11.5 (± 1.5)	n/a	0 (± 0)	n/a	n/a	n/a

+ paired t-test for IOP at various times post-OP compared to pre-OP;
* Wilcoxon Signed Rank Test for medication at various times post-OP compared to pre-OP;
++ t-Test for IOP between pseudophakic and combined group at various times
** Mann–Whitney U test for medication between pseudophakic and combined group at various times
