# Supplementary material for: Clinical outcomes of Preserflo MicroShunt implant procedure in open‑angle glaucoma patients - a 1000 eyes case-series; including subanalysis of combined cataract-glaucoma surgery and standalone surgery in pseudophakic eyes
Source: Graefes Arch Clin Exp Ophthalmol. 2026 Mar 28;264(10):3069–80. doi: 10.1007/s00417-026-07199-z (PMC13635017; doi:10.1007/s00417-026-07199-z)
Supplement: Supplementary file 2 [file 417_2026_7199_MOESM2_ESM.pdf]

Supplementary table 2

Intra ocular pressure (IOP) and glaucoma medication in phakic eyes: follow-up to 6 years after surgery.

	all eyes							
**Time after surgery**	No. eyes	Median	IOP - mean (mmHg ± SD)	p-Value+	Med. - mean (No.)	p-Value*	IOP p-Value#	Med. p-Value#
pre-surgery	391	23	24.7 (± 8.3)	-	2.54 (± 1.13)	-	0.39	0.01
1 day	228	9	9.4 (± 3.7)	<0.01	-	-	0.06	-
1 week	314	10	11.0 (± 5.5)	<0.01	0.00 (± 0.00)	<0.01	<0.01	0.01
1 month	311	10	11.7 (± 5.1)	<0.01	0.04 (± 0.23)	<0.01	<0.01	0.62
1/4 year	285	11	12.0 (± 4.9)	<0.01	0.06 (± 0.33)	<0.01	0.05	0.37
1/2 year	213	12	12.8 (± 4.8)	<0.01	0.07 (± 0.36)	<0.01	0.59	0.25
1 year	180	12	13.1 (± 4.3)	<0.01	0.14 (± 0.55)	<0.01	0.42	0.78
1.5 years	139	12	12.8 (± 3.6)	<0.01	0.11 (± 0.46)	<0.01	0.72	0.21
2 years	127	12	12.6 (± 2.9)	<0.01	0.19 (± 0.66)	<0.01	0.23	0.68
2.5 years	113	12	12.9 (± 2.8)	<0.01	0.13 (± 0.59)	<0.01	0.28	0.79
3 years	89	13	13.6 (± 5.9)	<0.01	0.25 (± 0.71)	<0.01	0.23	0.59
4 years	46	14	14.0 (± 3.0)	<0.01	0.24 (± 0.60)	<0.01	0.47	0.48
5 years	19	14	14.8 (± 3.4)	<0.01	0.11 (± 0.31)	<0.01	0.53	0.34
6 years	2	14	14.0 (± 2.0)	0.36	0.67 (± 0.47)	0.18	0.31	0.63

+ paired t-test for IOP at various times post-OP compared to pre-OP;

* Wilcoxon Signed Rank Test for medication at various times post-OP compared to pre-OP;

ANOVA-analysis: combined vs. standalone pseudophakic vs. standalone phakic group at various times
