# Supplementary material for: Clinical outcomes of Preserflo MicroShunt implant procedure in open‑angle glaucoma patients - a 1000 eyes case-series; including subanalysis of combined cataract-glaucoma surgery and standalone surgery in pseudophakic eyes
Source: Graefes Arch Clin Exp Ophthalmol. 2026 Mar 28;264(10):3069–80. doi: 10.1007/s00417-026-07199-z (PMC13635017; doi:10.1007/s00417-026-07199-z)
Supplement: Supplementary file 3 [file 417_2026_7199_MOESM3_ESM.pdf]

Supplementary table 3

No. of patients with secondary glaucoma surgery (due to inadequate device function, and other than bleb revision or needling).

	**glaucoma classification**			**Surgical procedure subgroups**		
**Time after surgery**	POAG	PEX	other	pseudophakic eyes	combined microshunt with cataract surgery	standalone procedure in phakic eyes
1 month	1	-	-	-	1	-
1/4 year	1	1	-	-	2	-
1/2 year	2	-	1	-	3	-
1 year	3	1	-	-	3	1
1.5 years	2	-	-	-	1	1
2 years	6	-	-	1	4	1
2.5 years	2	3	-	-	2	3
3 years	2	4	-	-	2	4
4 years	3	1	-	-	4	-
5 years	1	1	-	1	-	1
6 years	-	-	-	-	-	-
sum	23	11	1	2	22	11
% of implanted eyes	**3%**	**4%**	**2%**	**2%**	**4%**	**3%**

POAG-primary open-angle glaucoma; PEX- Pseudoexfoliative glaucoma
